# Obesogenic Memory Maintains Adipose Tissue Inflammation and Insulin Resistance

**DOI:** 10.20900/immunometab20200023

**Published:** 2020-06-15

**Authors:** Alecia M. Blaszczak, Matt Bernier, Valerie P. Wright, Gina Gebhardt, Kajol Anandani, Joey Liu, Anahita Jalilvand, Stephen Bergin, Vicki Wysocki, Arpad Somogyi, David Bradley, Willa A. Hsueh

**Affiliations:** 1 Diabetes and Metabolism Research Center, Division of Endocrinology, Diabetes, and Metabolism, Department of Internal Medicine, Wexner Medical Center, Columbus, OH 43210, USA; 2 Campus Chemical Instrument Center, Mass Spectrometry and Proteomics, The Ohio State University, Columbus, OH 43210, USA

**Keywords:** obesity, weight loss, visceral adipose tissue, inflammation

## Abstract

**Background:**

Obesity is characterized by visceral adipose tissue (AT) inflammation. Immunosuppressive regulatory T cells (Tregs), phagocytic M2-like macrophages, and innate lymphoid cells type 2 (ILC2) control lean AT inflammation to maintain systemic insulin sensitivity, while the loss of these cells in obesity leads to AT inflammation and insulin resistance (IR).

**Objective:**

The objective of this study was to determine if weight loss following obesity would correct AT inflammation and systemic metabolism.

**Results:**

After six months of high fat diet (HFD) in male C57/Bl6 mice, flow analyses of epidydimal AT stromal vascular fraction (SVF) revealed depleted Tregs by 50%, doubling of CD8^+^ T cells, tripling of pro-inflammatory M1-like macrophages, and an 80% drop in ILC2 cells associated with changes in pro-inflammatory adipocyte and macrophage gene expression. Despite normalization of body weight, fat, and adipocyte size, mice ingesting 3 months of high-fat diet (HFD) followed by 3 months of chow-diet remained more insulin resistant and glucose intolerant than chow-fed animals. Adipocytes, AT Tregs, CD8^+^ T cells, ILC2 cells, and M1-like macrophages all failed to normalize with weight loss.

**Conclusions:**

Persistent AT inflammation contributes to the maintenance of IR despite body weight and fat normalization in previously obese mice. These findings highlight the importance of obesity prevention to avoid the consequences of “obesogenic memory.”

## INTRODUCTION

Obesity is a worldwide epidemic associated with increased morbidity and mortality leading to a major emphasis on weight loss [1]. Inflammation is a key component of AT expansion [2,3] and contributes to multiple complications of obesity, particularly insulin resistance (IR) and type 2 diabetes mellitus (T2DM) [4]. In lean mouse AT, an anti-inflammatory state [5] is orchestrated through the interaction of T cells, primarily immunosuppressive regulatory T cells (Tregs) and CD4^+^ Th2 cells [6], M-like2 macrophages [7], innate lymphoid cells type 2 (ILC2) [8] and eosinophils [9]. Tregs function as important immunomodulatory cells through the release of immunosuppressive cytokines including TGFβ and IL10 and promote an increase in anti-inflammatory immune cell subsets including M2-like macrophages and ILC2 cells which further support Treg differentiation and maintenance [10,11]. During high-fat diet (HFD), the AT microenvironment shifts to a pro-inflammatory milieu characterized by an increase in pro-inflammatory T cells including CD4^+^ Th1 and cytotoxic CD8^+^ T cells [12], a profound drop in immunosuppressive Tregs, and a switch to M1-like macrophages [13]. We found that adipocytes, through adaptive immune mechanisms, are responsible for the obesity-related increase in AT CD4^+^ Th1 cells and decrease in Tregs [14,15]. This drop in AT Tregs has important metabolic implications, specifically contributing to systemic IR. Obese humans [16] and mice [6] reveal decreased numbers of AT Tregs. Use of a CD3 depleting antibody in C57Bl/6 mice which promotes the expansion of the Treg population and depletion of all other CD3^+^ cells results in improvements in glucose tolerance [17]. In contrast, administration of an anti-CD25 antibody to deplete Tregs in db/db mice leads to worsening IR, increased visceral AT inflammation, and worsening of diabetic nephropathy; whereas repletion of Tregs in db/db mice improves insulin sensitivity and diabetic nephropathy, suggesting a role for AT Tregs in diabetic complications [18]. AT macrophages (ATM) are also determinants of systemic insulin action. Genetic depletion of cytokines in macrophages or prevention of conversion of phagocytic M2-like to pro-inflammatory M1-like macrophages attenuates HFD-induced IR [19–22].

Only recently have weight loss studies begun to examine AT immune cell changes. Indeed, weight loss following HFD is associated with residual IR and AT inflammation, but in most of these studies, body weight and fat did not return to lean control levels. Therefore, we hypothesized that HFD in C57Bl/6 mice followed by weight loss to levels in lean age-matched mice would normalize the AT immune cell population.

## MATERIALS AND METHODS

### Mouse Studies

**Figure 1.**
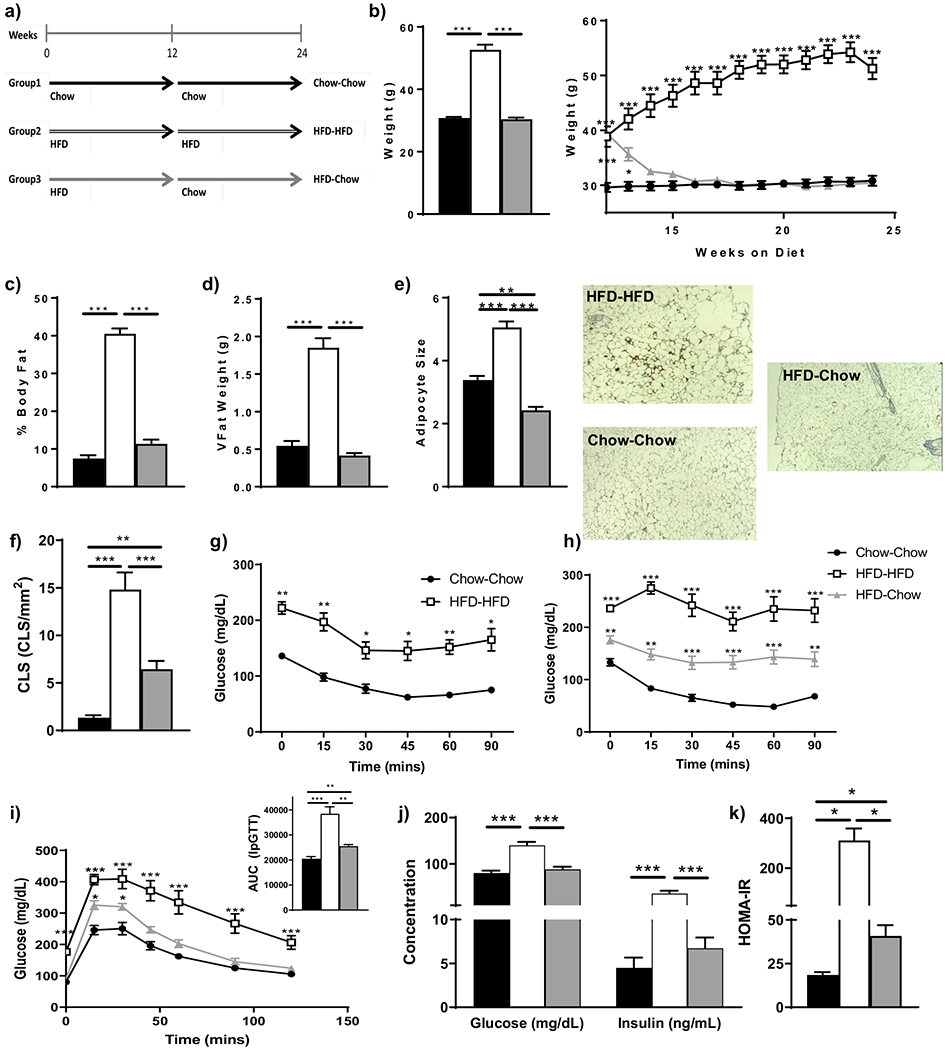
Effect of various diets on (**a**) C57/Bl6 mice at 24 weeks post-diet (*n* = 5/group) on (**b**) body mass, (**c**) adiposity, (**d**) weight of visceral fat at the time of sacrifice, (**e**) adipocyte size with representative H&E stained histological slides and (**f**) crown-like structures. Further metabolic analysis examined insulin sensitivity and glucose tolerance using the intraperitoneal insulin tolerance test with comparison to the chow-chow group for statistical analysis (**g**) prior to and after (**h**) diet change, (**i**) intraperitoneal glucose tolerance test with comparison to the chow-chow group for statistical analysis with associated area under the curve (AUC), and (**j**) circulating insulin and glucose and (**k**) HOMA-IR. * *p* < 0.05, **p < 0.01, or *** *p* < 0.001.

Male aged-matched C57/Bl6 mice (Jackson Laboratories) were placed on 60% HFD (Research Diets, Cat# D12492) at 6 weeks of age or maintained on chow (Teklad Diets, Cat# 8904) for 12 weeks. After 12 weeks, mice were either maintained on chow or HFD or switched to chow for the remaining 12 weeks (Figure 1a). Animals were grouped, housed and maintained on a 12-h light-dark cycle in pathogen-free facilities at the Ohio State University. At the end of the diet, mice underwent insulin tolerance testing (ITT) and intraperitoneal glucose tolerance testing (IpGTT), and measurement of body weight, adiposity and circulating levels of insulin and glucose. After 6 h fast (9 am–2 pm) for ITT or overnight fast (5 pm–9 am) for IpGTT, insulin (0.5 U/kg) or glucose (1 g/kg) was administered via intraperitoneal injection at T0 after obtaining fasting blood samples [14,15]. All experiments were approved by and in accordance with The Ohio State University Institutional Animal Care and Use Committee (2014A00000108-R1, approval date: 09/21/2017).

### Adipose Tissue Cell Isolation and Gene Expression Analyses

The epididymal fat pad was harvested and processed with collagenase digestion to isolate the adipocyte fraction and the stromal vascular fraction (SVF) [23]. Purified adipocytes were utilized for qtPCR gene expression with the addition of TriReagent. SVF was used for flow cytometry analysis and cell isolation by magnetic beads.

From the SVF, adipose tissue macrophages (ATM) and adipose resident T (ART) cells were sequentially isolated using biotinylated antibodies for F4/80 (eBioscience (Waltham, Massachusetts, USA), 13-4801-85, 1:200 dilution) and CD3e respectively (eBioscience (Waltham, Massachusetts, USA), 13-0031-85, 1:100 dilution). Cells were then incubated in the presence of streptavidin magnetic beads and placed on a magnetic stand for cell isolation as previously described [15]. Isolated beads were resuspended in TriReagent for downstream gene expression analysis.

For all qtPCR related experiments, RNA was extracted using the Direct-zol RNA MiniPrep kit (Zymo Research, Irvine, California, USA). cDNA was generated using the High-Capacity cDNA Reverse Transcription Kit (ThermoFisher Scientific, Waltham, Massachusetts, USA). For all gene expression analyses, primers and probes were purchased from Sigma or IDT in order to examine the targets of interest identified within this paper including genes related to inflammatory processes, MHCII components, and macrophage, T cell, and adipocyte defining genes. For analyses, all gene expression values were normalized to *Ppia* and compared to the chow-chow group.

### Flow Cytometry Analysis

The SVF from the digested adipose tissue or spleen were resuspended in FACS buffer and incubated for 10 min with the CD16/32 blocking antibody (BioLegend, Cat#101302). 100 μL of the resuspended cells were added to each flow tube. After the incubation, the surface antibodies were added and incubated at 4 °C in the dark for 30 min along with all necessary controls. The following fluorochrome-conjugated antibodies were used for cell surface identification of T cells and were purchased from BioLegend (San Diego, California, USA) unless otherwise noted: CD4 (Cat#100406), CD3 (Cat#100220), CD25 (Cat#101910), and CD45 (Cat#103116). For macrophage flow surface staining, the following fluorochrome-conjugated antibodies were used: CD45 (Cat#103116), CD11c (Cat#117327), and F4/80 (eBioscience, Cat#48-4801-82, Waltham, Massachusetts, USA). For ILC2 cell staining, the following antibodies were used: CD45 (Cat#103116) CD3 (Cat#100220), CD4 (Cat#100406), ST2 (Cat#145312), and CD25 (Cat#101910). Following the incubation, cells were washed two times with 2 mL of FACS buffer (1st wash) and 2 mL of PBS (2nd wash) and spun at 500 × *g* for 6 min at 4 °C. The cells were then resuspended in 500 μL of PBS with 0.5 μL of Fixable Viability Dye (ThermoFisher Scientific, Waltham, Massachusetts, USA, Cat#: L34962) for 30 minutes in the dark at 4 °C. After this incubation, cells were washed twice in FACS buffer as noted above. Following the washing steps for further T cell characterization, the eBioscience Foxp3 intracellular staining kit (Waltham, Massachusetts, USA (Cat#: 00-5523-00) was used following the manufacturer’s protocol along with the following intracellular stain used for Treg cell subset identification: FOXP3 (Cat# 126404). After the hour-long incubation with the intracellular antibody, cells were washed twice with permeabilization buffer from the eBioscience kit and resuspended in a final volume of 150 μL. All samples were run on the BD LSRII Flow Cytometer, and all analyses were performed using Flowjo software (Treestar, Ashland, Oregon, USA). The following gating structure was used for all cellular analyses: single cells (FSC-A vs SSC-A followed by FSC-H vs FSC-A) followed by live and immune cell gating (Fixable Viability Dye^+^ CD45^+^). For complete immune cell subset identification and flow gating strategies, see Supplemental Table S1 and Supplemental Figure S1 respectively.

### Blood Measurements

Blood was drawn (after a 6 h fast) into purple top EDTA tubes, spun at 2000 × *g* for 10 min, and the plasma collected and immediately placed in a −80 °C freezer. Glucose levels were measured using a Contour Next Easy meter, and insulin levels were measured in duplicate by the University of Cincinnati Medical Center MMPC (GRANT U2C DK059630).

### Adipocyte Size and Crown-like Structure Determination

Adipocyte size was measured using five images per animal on the Echo Revolve Microscope at 10× magnification (San Diego, California, USA). The image was divided into quartiles and a random number generator was used to select the quartile for quantification. The area of the quartile was measured through area tracing and the number of adipocytes counted for the given area. The average adipocyte size was determined by dividing the total number of adipocytes within the quartile by the quartile area.

For CLS determination, tissues were processed, embedded and F4/80 stained. Images were taken at 10× magnification. The image was divided into quartiles and a random number generator was used to select the quartile for CLS and area quantification. The average CLS was determined by dividing the total number of CLS within the quartile by the quartile area.

### Treg Differentiation Assay

Splenic naïve CD4 T cells from transgenic OTII (Jax: B6.Cg-Tg(TcraTcrb)425Cbn/J) mice were isolated following the manufacturer’s protocol (BioLegend MojoSort Mouse CD4 Naïve T Cell Isolation Kit, Cat#: 480039). Adipocytes of HFD-HFD, chow-chow, and HFD-chow animals were incubated with naïve splenic CD4 T cells isolated from OTII mice in the presence of ovalbumin. Recombinant interleukin (IL) 2 (5 ng/mL) and transforming growth factor beta-1 (TGFb1) (5 ng/mL) was added to the culture media to preferentially induce Treg differentiation [24]. After 4 days, T cells were collected and Tregs measured by flow cytometry.

### Statistical Analyses

All statistics and figures and graphs were created with Prism 6.0 software (GraphPad, San Diego, California, USA). One-Way ANOVAs were performed with sample sizes indicated in each figure. Values are expressed as mean ± SE, and the significance was set at a *p*-value < 0.05.

## RESULTS

### Loss of Weight and Body Fat after HFD Does Not Normalize Insulin Sensitivity and Glucose Tolerance

After 24 weeks of HFD (HFD-HFD) (Figure 1a), mice gained weight, and body fat and adipocyte size and crown-like structures (CLS) increased. At 12 weeks HFD-fed mice were switched to chow for 12 weeks (HFD-chow) (Figure 1a) at which time they exhibited reduced body weight, % body fat, visceral fat weight, identical to that of mice maintained on chow diet (chow-chow) and even lower adipocyte size and increased CLS (Figure 1b–f). As expected, before diet change, the HFD animals were more insulin resistant as compared to the chow-fed controls (Figure 1g). Despite the loss of weight and body fat with a switch to the chow diet, HFD-chow mice remained more insulin resistant and glucose-intolerant compared to mice never exposed to HFD (chow-chow) (Figure 1h–k).

### AT Tregs Increase with Weight Loss but CD8 T cells Remain Elevated

**Figure 2.**
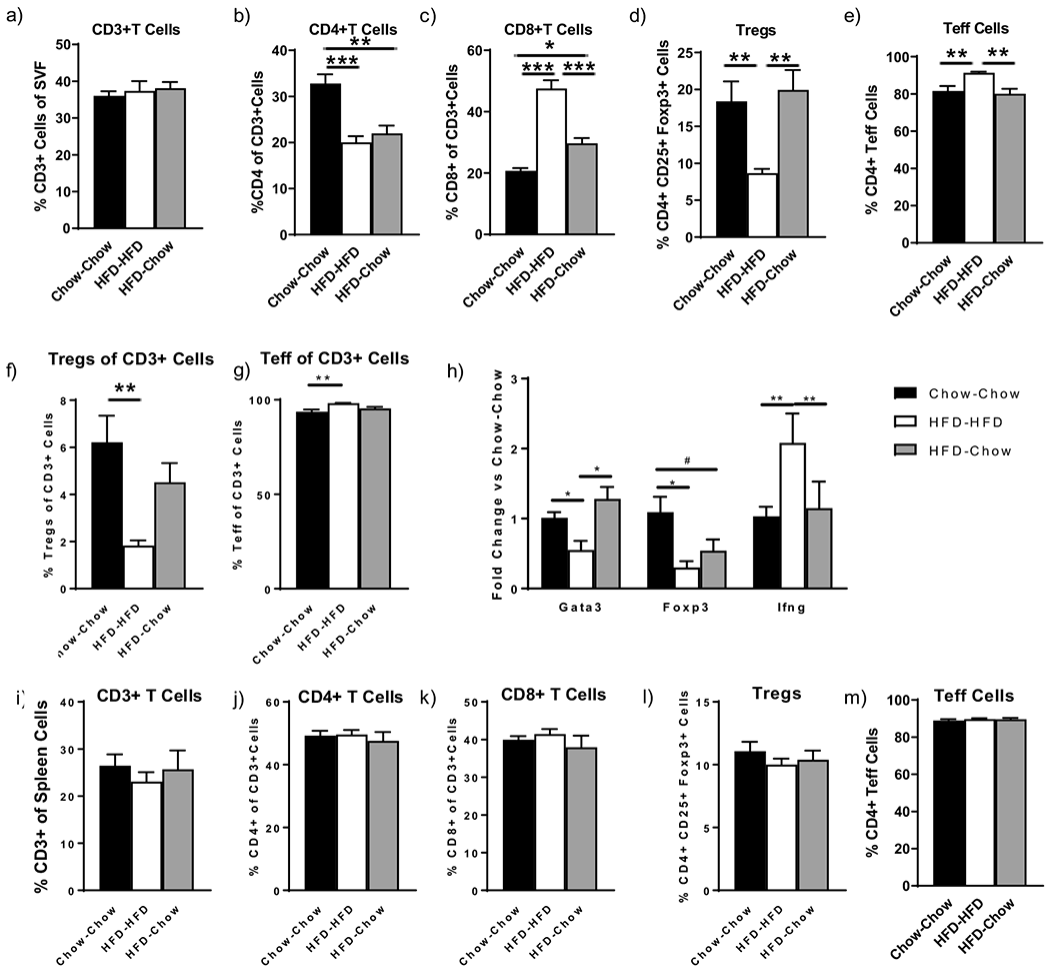
Effect of various diets on C57/Bl6 mice (*n* = 5/group) at 24 weeks post-diet on visceral adipose tissue (**a**) CD3^+^ T cells, (**b**) CD4^+^ T cells, (**c**) CD8^+^ T cells, (**d**) Tregs, (**e**) Teff cells and (**f**) Tregs and (**g**) Teff as a percent of CD3^+^ cells by flow cytometry and by (**h**) gene expression data on CD3^+^ bead isolated T cells. Changes in spleen: (**i**) CD3^+^ T cells, (**j**) CD4^+^ T cells, (**k**) CD8^+^ T cells, (**l**) Tregs and (**m**)Teff cells. # *p* < 0.10, * *p* < 0.05, ** *p* < 0.01, or *** *p* < 0.001.

In the epididymal fat pad, there was no change in CD3^+^ cells as a %SVF (defined as all live cells after AT digestion, density separation, and red blood cell lysis) during the different dietary manipulations (Figure 2a). However, CD4^+^ T cells decreased as a % of CD3^+^ cells in HFD-HFD mice but did not increase in the HFD-chow mice (Figure 2b). CD8^+^ T cells increased in HFD-HFD mice but did not return to baseline in HFD-chow mice (Figure 2c). Thus, the T cell distributions remained pro-inflammatory despite weight loss. Tregs as a % CD4^+^ cells decreased with HFD and increased with weight loss (Figure 2d); however, as a percent of CD3^+^ T Cells (Figure 2f), they were not different from the HFD-HFD or chow-chow animals suggesting an incomplete normalization. In contrast, T effector cells (CD4^+^ Foxp3−) increased in the HFD-HFD animals and returned to chow-chow levels after weight loss (Figure 2e).

Expression of *Ifnγ*, produced by CD8^+^ and CD4^+^ Th1 cells increased in the HFD-HFD animals in bead-isolated CD3^+^ T cells from SVF and returned to chow-chow levels with weight loss. In contrast, *Gata3*, the primary marker of Th2 T cells trended to decrease with HFD and increased after the switch to a chow diet (Figure 2h). However, the expression of *Foxp3*, a marker of Tregs, decreased with HFD but did not return to chow-chow levels with weight loss (Figure 2h) consistent with the Treg flow analyses (Figure 2f). No differences between T cell populations were noted in the spleen (Figure 2i–m), suggesting no changes in peripheral T cells among the diet groups.

### Cd11c^+^ Macrophages Remain Elevated despite Weight Loss

**Figure 3.**
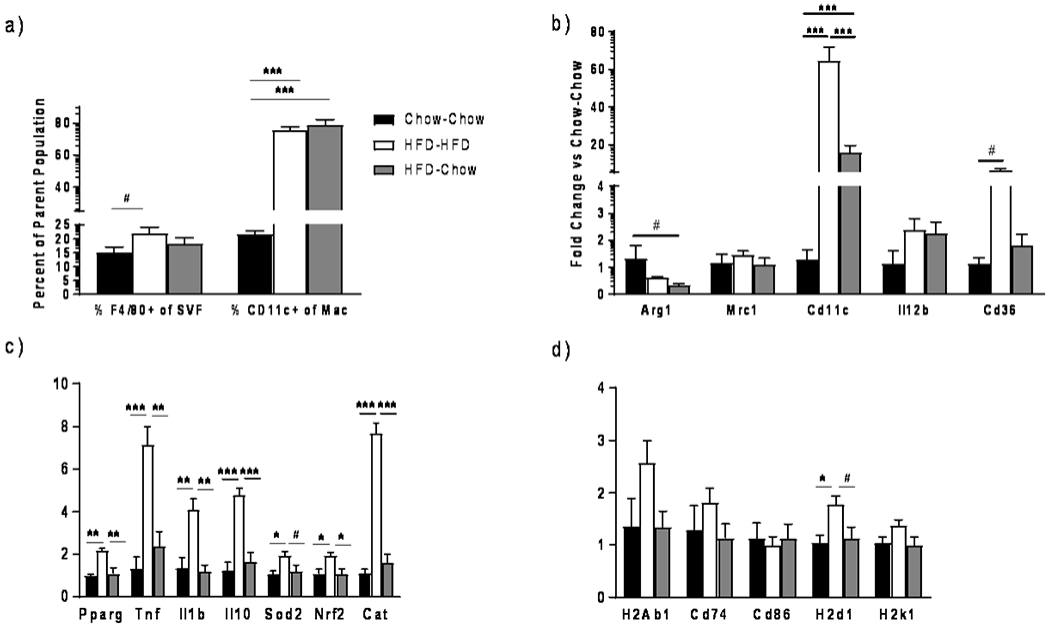
Effect of various diets on C57/Bl6 mice (*n* = 5/group) at 24 weeks post-diet on visceral adipose tissue macrophages by (**a**) flow cytometry and by qtPCR on (**b**) macrophage markers, (**c**) inflammation markers and (**d**)MHCII and MHCI related genes. # *p* < 0.10, * *p* < 0.05, ** *p* < 0.01, or *** *p* < 0.001.

Cd11c (*Itgax*) is considered a marker of M1-like macrophages in mice, while *Arg1* and *Mrc1* are markers of phagocytic M2-like macrophages [13]. Flow analyses revealed that macrophage abundance increased in SVF of HFD-HFD vs. chow-chow mice (Figure 3a), but was not different between HFD-HFD and HFD-chow mice. The %CD11c macrophages markedly increased in HFD-HFD vs chow-chow SVF and remained elevated in HFD-chow SVF suggesting a persistence of the inflammatory M1-like state. This observation was supported by gene expression of the bead-isolated ATMs; *Itgax* (Cd11c) expression increased during HFD-HFD and decreased in the HFD-chow group, but remained substantially above chow-chow levels (Figure 3b). *Mrc1* expression did not change, whereas *Arg1* trended to decrease in the weight loss mice. Thus, M2-like macrophage markers do not increase and M1-like markers do not decrease to lean values following weight loss. However, expression of genes related to cytokines (*Tnf, Il1b, Il10*) and oxidative stress (*Nrf1*, *Sod, Cat*), and *Ppary*, all increased during HFD-HFD and decreased during HFD-chow (Figure 3c). Lastly, we assessed genes involved in adaptive (*H2Ab1*, *Cd74*, *Cd86*) and innate immunity (*H2d1*, *H2k1*). There was a trend for major histocompatibility complex I and II (MHCI/II) genes (*H2Ab1*, *CD74*, *H2k1*) to increase with HFD and decrease during HFD-chow which was significant for *H2d1* (Figure 3d). Thus, ATM normalization of cytokine and antigen presentation genes occurs with weight loss.

### ILC2 Cells Remain Depleted with Weight Loss and Correlate with Glucose Intolerance

**Figure 4.**
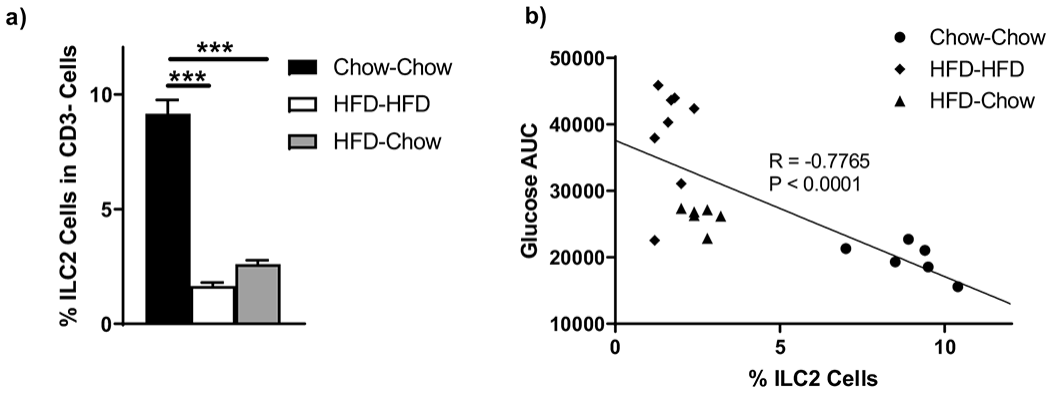
Effect of various diets on C57/Bl6 mice (*n* = 8/group for HFD-HFD and *n* = 6 for chow-chow and HFD-chow) at 24 weeks post-diet on visceral adipose tissue (**a**) ILC2 cells as a percent of CD3^−^ cells and their relation to (**b**) glucose area under the curve. *** *p* < 0.001.

HFD animals demonstrated depletion of visceral AT ILC2 cells (Figure 4a) which did not recover after 12 weeks of weight loss. Previous data has shown that ILC2 cells in mice contribute to insulin resistance [25], and in our HFD-HFD and HFD-chow mice, there was a strong negative correlation between IpGTT glucose AUC and % ILC2 cells (Figure 4b). Taken together, these results suggest that ILC2 cells may be a key factor in determining glucose tolerance and support previous reports suggesting they maintain AT Tregs [26].

### Innate and Adaptive Pro-Inflammatory Genes Increase during HFD and Decrease from HFD to Chow in Visceral Adipocytes

**Figure 5.**
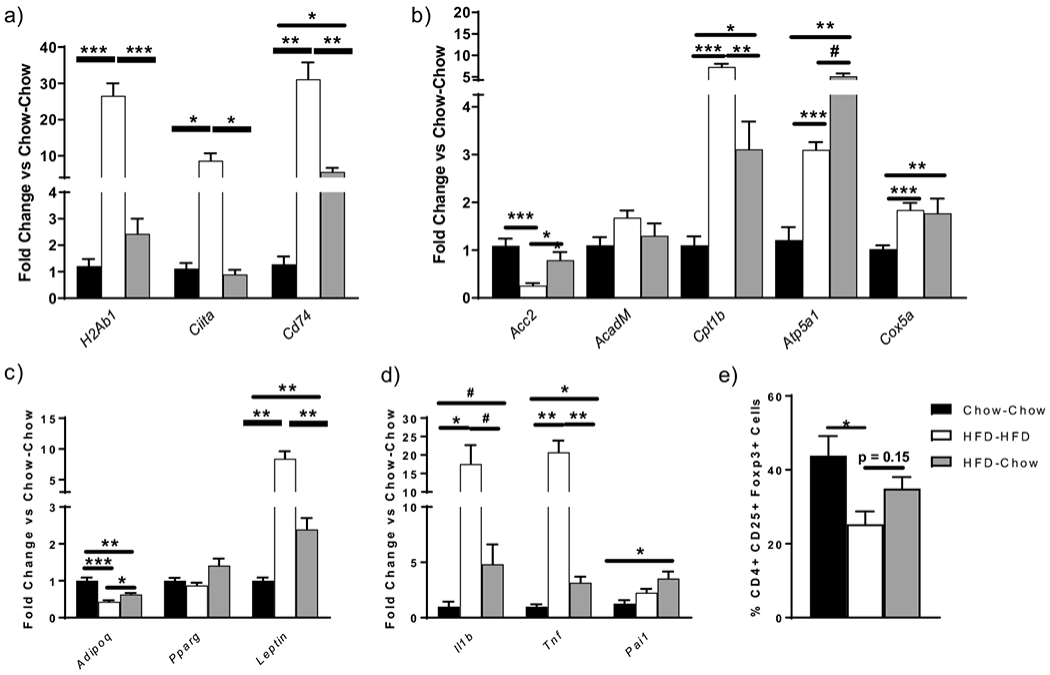
Effect of various diets on C57/Bl6 mice (*n* = 5/group) at 24 weeks post-diet on visceral adipocyte gene expression for genes related to (**a**) the MHCII pathway, (**b**) metabolism, (**c**) adipokines and (**d**) inflammation. For further analysis, VAd cells were isolated 24 weeks post-diet (*n* = 5/group) and were co-cultured with splenic naïve CD4 T cells from transgenic OTII mice for four days in triplicates in the presence of OVA peptide, IL2 and TGFbeta to stimulate Treg differentiation. T cells were then collected and subjected to (**e**) flow cytometry analyses. # *p* < 0.10, * *p* < 0.05, ** *p* < 0.01, or *** *p* < 0.001.

Adipocyte MHCII genes increased in HFD-HFD and decreased in HFD-chow, although *Cd74*, a key regulator of the MHCII pathway remained elevated (Figure 5a). As expected, adiponectin decreased in the HFD-HFD vs chow-chow group and increased in the HFD-chow group but not to chow-chow levels, while leptin increased in the HFD-HFD group and decreased in the HFD-chow group, but not fully to levels in chow-chow mice (Figure 5c). Cytokine expression *Il1b* and *Tnf*) increased during HFD-HFD and decreased but not to chow-chow levels following weight loss (Figure 5d). Thus, sustained adipocyte inflammation marked by the incomplete restoration of adiponectin and leptin expression as well as pro-inflammatory cytokine markers *Il1b* and *Tnf* characterized weight loss animals. Adipocyte metabolic genes changed with HFD and also did not normalize. *Cpt1b, Cox5a*, and *Atp5a1* dramatically increased with a decrease in expression of *Acc2*, but these genes did not normalize with weight loss (Figure 5b). Thus, adipocyte metabolism is shifted in obesity with a reduction in fatty acid synthesis and an increase in mitochondrial and in β oxidation genes, which persists despite weight loss.

### Treg Generation Is Reduced in Co-Culture Using Adipocytes Isolated from HFD-HFD Animals but Does Not Completely Normalize with Weight Loss

Adipocytes are antigen-presenting cells capable of generating CD4^+^ Th1 cells and Tregs [14,15]. Given the decrease in AT Tregs, we sought to examine if alterations in adipocyte antigen presentation could account for the sustained depletion of Tregs with weight loss. The % Tregs generated in co-culture with visceral chow-chow adipocytes under Treg promoting conditions was ~45%, but reduced to nearly 20% with HFD-HFD adipocytes of equal volumes, similar to previously reported data [14]. However, adipocytes isolated from HFD-chow animals only partially increased Treg generation compared to adipocytes isolated from chow-chow mice, as indicated by the lack of difference noted between the HFD-chow and either the HFD-HFD or chow-chow mice with increased Treg generation (Figure 5e). Thus, despite decreased inflammatory gene expression, these results suggest that the adipocyte antigen-presenting capacity did not decrease completely despite weight loss (Figure 2).

## DISCUSSION

Weight loss is universally advocated to prevent and treat the inflammatory-induced complications of obesity including IR and T2DM. However, our findings suggest that obesity results in residual inflammation in nearly all AT compartments in comparison to pre-HFD levels. These persistent changes likely contribute to maintained IR and abnormal glucose metabolism, despite decreased body weight and % body fat to that of lean controls and decreased adipocyte size to less than lean controls. Thus, AT “obesogenic memory” complicates weight management, so prevention of obesity or early intervention before major AT immune cell changes is critical. In addition, future research to elucidate mechanisms causing the persistent adipose tissue inflammation despite weight loss is important so that treatment strategies can be developed to quell this inflammation and aid in the prevention of obesity-associated complications.

Pro-inflammatory changes in AT immune cells during obesity regulate IR. HFD leads to adipose macrophage invasion with a switch from phagocytic M2-like to pro-inflammatory M1-like macrophages [13,27]. Prevention of macrophage migration [28], the M2-like to M1-like switch [21], or knockout of macrophage inflammatory factors [19,22] attenuates HFD-induced IR without affecting body weight, highlighting an AT macrophage role in IR. Subsequently, a series of elegant adipose resident T cell (ART) studies demonstrated an increase in AT CD8^+^ T cells [12] and CD4^+^ Th1 cells [29] and a decrease in Tregs and ILC2 cells which contributes to IR [6,15,17,18]. Adoptive transfer of Tregs [17] or ILC2 cells [8] attenuated HFD-induced IR, underscoring the importance of these cells in regulating systemic metabolism.

We, therefore, interrogated which changes in AT immune cells could explain the lack of improvement in insulin sensitivity and glucose tolerance in weight loss animals. As expected, macrophage abundance was increased in the HFD-HFD versus chow-chow groups with a marked increase in CD11c^+^ macrophages in the HFD-HFD group that did not substantially decrease in the HFD-chow group. Consistent with flow analyses, *Cd11c* expression levels were markedly elevated in HFD-HFD mice compared to chow-chow with intermediate levels seen in HFD-chow mice. The expression of the M2-like marker *Arg1* did not change. Thus, M1-like macrophage biomarkers continue to predominate in AT despite weight loss. Moreover, CLS, comprised of macrophages and other immune cells decreased with weight loss, but not to chow-chow levels. In contrast, weight loss completely attenuated the increased macrophage expression of inflammatory cytokines and antioxidant response (*Nrf2, Sod2*, and *Cat*), which reflect HFD-associated oxidative stress.

In the adipocyte, the expression of inflammatory markers changed with weight loss. *Adiponectin* decreased and *leptin*, *Tnf* and *Il-1b* increased in the HFD-HFD group, as expected, but did not normalize with weight loss; the lack of normalization of these important adipokines likely contributes to residual inflammation. Expression of genes involved in fatty acid metabolism showed the suppression of lipid synthesis and storage (*Acc2*) and increased fatty acid utilization (*Cpt1B*, *Atp5a1*, *Cox5a*), which may be an attempt to combat the increased fatty acid intake. These processes only partially reversed with weight loss and could lead to elevated circulating lipids, a known contributor to IR [30]. In contrast, we previously reported that antigen presentation, mediated through the major histocompatibility complex II (MHCII) pathway, increases as early as 2 weeks of HFD resulting in increased AT CD4^+^ Th1 cells and decreased AT Tregs [15]. Genetic loss of adipocyte MHCII attenuates IR and AT T cell changes, indicating that the adaptive immune response of the adipocyte significantly contributes to AT inflammation [14]. There was a complete reversal of *Ciita*, the major transcriptional regulator of the MHCII pathway, and *H2Ab1*, one of the arms, but *Cd74*, which enables the MHCII pathway, remained elevated.

In obese mice, AT ILC2 cells were depleted, but substantially failed to recover with weight loss and correlated with sustained glucose-intolerance. Previous reports show that loss of ILC2 cells in Rag1−/− mice leads to worsening systemic metabolism with a corresponding increase in body weight, whereas supplementation of ILC2 cells in obese mice leads to weight loss and improved metabolism [8]. It has also been reported that ILC2 cells support Tregs, consistent with our finding that Tregs do not normalize in our weight loss model [10].

Lastly, CD4^+^ T cells and Tregs as a percent of CD3^+^ T cells decreased and CD8^+^ T cells increased with HFD; none returned to chow-chow levels with weight loss consistent with greater T cell inflammation. Taken together, these data suggest that macrophages, T cells, ILC-2 cells, and adipocytes retain components of their pro-inflammatory state to promote AT inflammation and abnormal glucose metabolism despite weight reduction.

Previous studies in mice found residual visceral AT inflammation after weight loss. One study did not see a drop in AT inflammation after 12 weeks HFD followed by 3 weeks low-fat diet with modest improvements in insulin sensitivity but body weight and fat did not return to pre-HFD levels, complicating interpretation of the inflammatory and metabolic changes [31]. Another study showed increased crown-like structures and increased AT inflammatory gene expression despite a return of weight, body fat, and adipocyte size to control chow levels; residual liver and adipose IR was still present, but specific AT immune cell contributions were not explored [32]. Other investigators found persistent elevations in CD4^+^ and CD8^+^ T cells despite a return of body weight and fat to levels seen in control diet mice; however, they did not assess pro- or anti-inflammatory CD4 T cells or macrophages [33]. Our macrophage changes resemble those reported by Zamarron et al. which showed persistence of CD11c^+^ macrophages despite a decrease in AT macrophages and increased ATM pro-inflammatory gene expression following weight loss [34], but body weight did not decrease to the weight of non-HFD challenged mice, even as long as 24 weeks of chow diet following 12 weeks of HFD. Similarly, in the study by Vatarescu et al. sustained changes were noted in AT macrophages despite weight loss although weight and epidydimal fat pad mass did not normalize to the chow-fed animals. In this study they also noted improvements in lipid clearance from the ATM and from the liver which is a potential mechanism for the improved, although not normal, glucose tolerance and insulin sensitivity in these animals [35]. A weight cycling study using 9 weeks of a 60% HFD followed by 4 weeks of a 10% low-fat diet suggested return of weight and metabolic parameters to pre HFD levels, although AT immune cells were not assessed after weight loss, and the second bout of HFD resulted in worse inflammation and metabolic changes than after the first exposure to HFD [36]. The authors noted that a limitation of their study was the use of the extremely low fat or calorie diet used for weight loss, which would not be employed by dieting humans [37]. Using a standard chow diet resulting in a slower weight loss, we did not find normalization of insulin sensitivity or glucose tolerance, despite the achievement of pre-HFD body weight and fat. Our comprehensive analyses suggest that AT adipocytes, T cells, and macrophages became less inflamed with weight loss, but do not return to levels seen in mice never been exposed to obesity. This lack of normalization could explain the heightened AT inflammation and IR seen with weight cycling [36].

“Trained immunity” is the persistent epigenetic [38] and metabolic reprogramming [39] of innate immune cells after pathogen exposure. Epigenetic reprogramming of these cells in the bone marrow is mediated by IL1β [40], a known AT inflammatory marker. In atherosclerotic prone Ldlr^−/−^ animals, *Christ et al.* recently demonstrated that western diet (high in fat and cholesterol) can be a “sterile” immune trigger and despite normalization of circulating inflammatory markers and serum cholesterol, splenic and bone marrow-derived monocytes as well as granulocyte-monocyte precursor cells retained their pro-inflammatory phenotype. Upon exposure to pathogens, these cells were primed allowing for a more robust inflammatory response which was partially hypercholesterolemia and NLRP3-dependent [41]. Given the persistent inflammation seen in adipocytes, ATMs, and ARTs in our weight loss model, investigation of trained immunity may provide novel insight into the mechanism by which these immune cells remain in a primed and inflammatory state even after body weight normalization. This mechanism may also prove to be important in the persistent and step-wise increase in AT inflammation noted in weight cycling.

In summary, we performed a comprehensive investigation of alterations in adipocyte immune and metabolic changes; ILC2 cells; ATM pro-inflammatory cytokines and M1/M2-like markers; and ART subtypes to determine the effect of weight normalization in obese mice. Importantly, loss of body weight and fat reached comparable levels to mice maintained on chow diet. HFD induced inflammation in all immune cells; however, residual proinflammatory changes remained after weight loss, that contributed to persistent IR and glucose-intolerance.

## CONCLUSIONS

These observations underscore the need for further study of early intervention in obesity to avoid potentially permanent AT inflammation that profoundly impacts systemic metabolism. In addition, these results highlight the potentially harmful consequences of weight cycling characterized by progressive increases in AT inflammation with each period of weight gain. Preemptive intervention should occur at the overweight stage before substantial weight gain and AT immunological shifts with efforts focused on avoiding obesity. Future research should also aim to elucidate the mechanisms behind the sustained AT inflammation despite weight loss. In doing so, treatments targeting these pathways can be developed in the hopes that they prevent the progression of the many inflammatory complications of obesity that lead to accelerated morbidity and mortality.

## Supplementary Material

supplementary material

## ABBREVIATIONS

ATM: Adipose Tissue Macrophage
ART: Adipose Resident T Cell
HFD: High-Fat Diet
ILC2: Innate Lymphoid Cell Type 2
IpGTT: Intraperitoneal Glucose Tolerance Test
IR: Insulin Resistance
ITT: Insulin Tolerance Test
MHCII: Major Histocompatability Complex II
SVF: Stromal Vascular Fraction
T2DM: Type 2 Diabetes Mellitus
Treg: Regulatory T Cell
