# Supplementary material for: Obesogenic Memory Maintains Adipose Tissue Inflammation and Insulin Resistance
Source: Immunometabolism. Author manuscript; Available in PMC 2020 Aug 6. (PMC7409818; doi:10.20900/immunometab20200023)
Supplement: supplementary material [file NIHMS1604415-supplement-supplementary_material.pdf]

Supplemental Table S1. Immune Cell Characterization by Flow Cytometry

Immune Cell	Characterization
Macrophage	CD45+F4/80+
M1-like Macrophage	CD45+F4/80+CD11c+ (as % mac)
CD3+ T Cell	CD45+CD3+
CD8+ T Cell	CD45+CD3+ CD8+ (as % CD3+)
CD4+ T Cell	CD45+CD3+ CD4+ (as % CD3+)
Teff Cell	CD45+CD3+ CD4+ CD25-FOXP3- (as % CD4+)
Regulatory T Cell (Treg)	CD45+CD3+ CD4+ CD25+FOXP3+ (as % CD4+)
ILC2	CD45+CD3-CD4-FOXP3-CD25+IL33R+

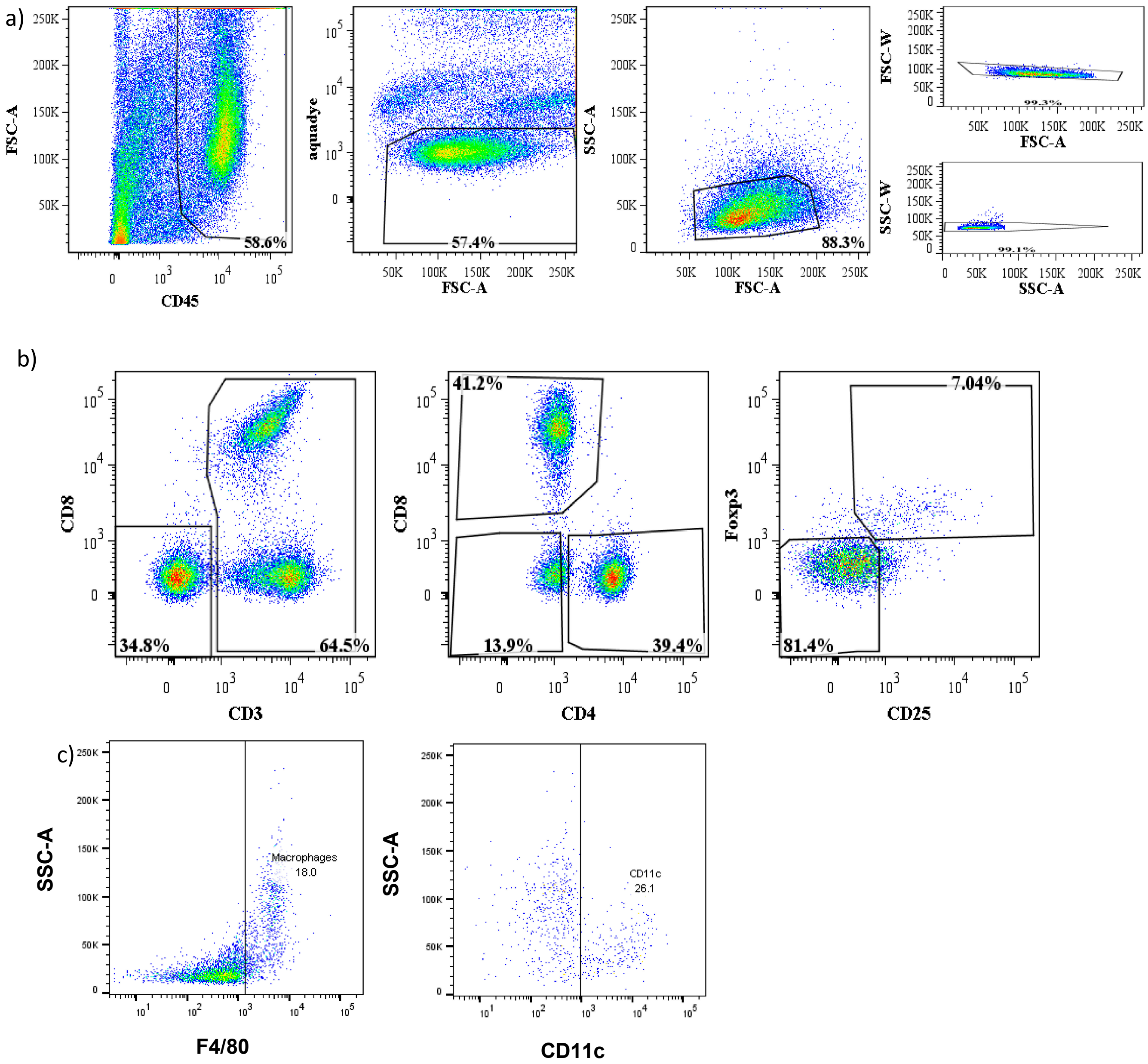

d)

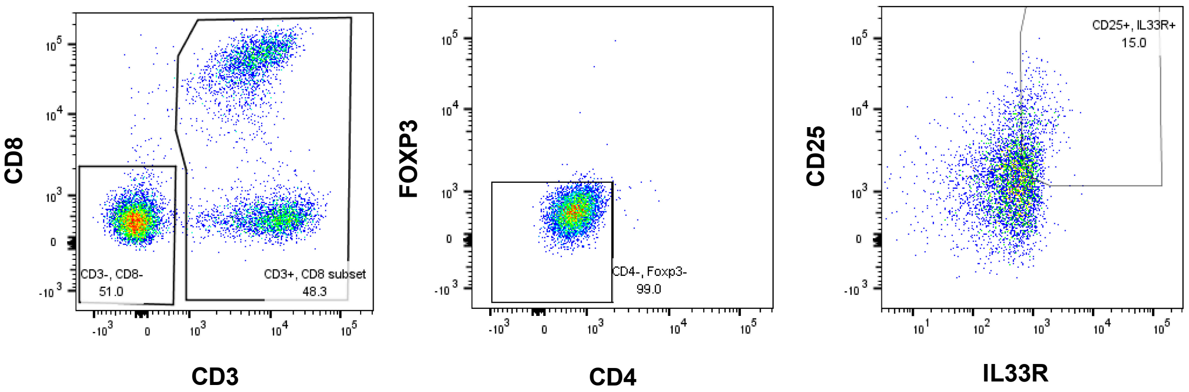

Supplemental Figure S1. Immune Cell Characterization with Representative Flow Cytometry Gating for a) all cells, b) T cells, c) macrophages, and d) ILC2 cells.
